# PP56 National Immunization Technical Advisory Groups Are Essential For Successful Implementation Of The European Health Technology Assessment Regulation

**DOI:** 10.1017/S0266462324002277

**Published:** 2025-01-07

**Authors:** Sharon Wolters, Ramesh Marapin, Jasmijn Beekman, Gabriel Gurgel, Anna Viceré

## Abstract

**Introduction:**

Starting from 2030, vaccines in the European Union (EU) require joint clinical assessments (JCAs), and joint scientific consultations (JSCs) can be requested from 2025. Involvement of national immunization technical advisory groups (NITAGs) is crucial to address vaccine specificities. However, NITAGs are currently not considered in the EU health technology assessment (HTA) framework. This study highlights potential risks for non-applicability of vaccine JCA reports nationally.

**Methods:**

This study examined where in the JCA and JSC implementation process NITAGs could play an important role and highlighted the practical challenges of incorporating vaccine JCA reports into already very heterogeneous and complex vaccine access pathways across EU Member States. The EU HTA Regulation process was tested for three countries with different vaccine market access characteristics. This study was conducted using JCA guidance documents, NITAG vaccine assessment guidelines, and communications with the respective decision-making bodies.

**Results:**

The EU HTA Regulation framework requires adjustments for vaccine-specific considerations. NITAGs across the EU vary in experience, resources, expertise, and influence on decision-making regarding vaccine assessment and inclusion in the national immunization program. In addition to JCA and JSC, the EU HTA framework also covers horizon scanning, which several NITAGs are currently involved with at national level.. However, the EU HTA framework currently lacks explicit requirements for NITAG input in horizon scanning, JSC, and JCA processes. This could lead to unnecessary duplication of work, further complexity of the processes, and lengthening of population time to access to new vaccines.

**Conclusions:**

The EU HTA framework of vaccines aims to avoid duplicate efforts and enhance patient access, but current processes that will be introduced may not achieve this optimally. Early and systematic inclusion of NITAGs in the horizon scanning, JSC, and JCA processes is pivotal to mitigate the risk of non-applicability and to successfully realize the objectives of the EU HTA framework.

